# Real-time Twitter interactions during World Breastfeeding Week: A case study and social network analysis

**DOI:** 10.1371/journal.pone.0249302

**Published:** 2021-03-29

**Authors:** Sara Moukarzel, Martin Rehm, Anita Caduff, Miguel del Fresno, Rafael Perez-Escamilla, Alan J. Daly

**Affiliations:** 1 Larsson-Rosenquist Foundation Mother-Milk-Infant Center of Research Excellence, University of California San Diego, La Jolla, CA, United States of America; 2 Department of Education Studies, University of California San Diego, La Jolla, CA, United States of America; 3 Institute of Educational Consulting, University of Education Weingarten, Weingarten, Germany; 4 Department of Social Work, National Distance Education University, Madrid, Spain; 5 Department of Social and Behavioral Sciences, Yale School of Public Health, New Haven, CT, United States of America; Universita degli Studi di Milano, ITALY

## Abstract

Using Twitter to implement public health awareness campaigns is on the rise, but campaign monitoring and evaluation are largely dependent on basic Twitter Analytics. To establish the potential of social network theory-based metrics in better understanding public health campaigns, we analyzed real-time user interactions on Twitter during the 2020 World Breastfeeding Week (WBW) as an exemplar case. Social network analysis (SNA), including community and influencer identification, as well as topic modeling were used to compare the activity of *n* = 29,958 campaign participants and *n* = 10,694 reference users from the six-months pre-campaign period. Users formed more inter-connected relationships during the campaign, retweeting and mentioning each other 46,161 times compared to 10,662 times in the prior six months. Campaign participants formed identifiable communities that were not only based on their geolocation, but also based on interests and professional background. While influencers who dominated the WBW conversations were disproportionally members of the scientific community, the campaign did mobilize influencers from the general public who seemed to play a “bridging” role between the public and the scientific community. Users communicated about the campaign beyond its original themes to also discuss breastfeeding within the context of social and racial inequities. Applying SNA allowed understanding of the breastfeeding campaign’s messaging and engagement dynamics across communities and influencers. Moving forward, WBW could benefit from improving targeting to enhance geographic coverage and user interactions. As this exemplar case indicates, social network theory and analysis can be used to inform other public health campaigns with data on user interactions that go beyond traditional metrics.

## Introduction

In today’s digital world, using social media has become an important strategy to support public health efforts and outcomes [1, 2]. Twitter, in particular, provides a unique source of big data, which supports monitoring, detecting, and forecasting disease outbreaks and health conditions (e.g., measles, Ebola, obesity, depression) as well as intervening through awareness campaigns [3, 4]. The uniqueness of Twitter stems from its increased popularity among hundreds of millions of diverse users across countries and world regions, the ability to capture activity in real-time, and the relative ease to access publicly available content [3]. Traditionally, analysis of Twitter activity has been limited to Twitter Analytics, a built-in data-tracking platform that provides basic impressions and engagement metrics (e.g., number of retweets, followers, mentions) [4]. However, recent advances in software analytics, grounded in social network theory, have made it possible to analyze Twitter data in much more nuanced, meaningful, and theoretically grounded ways [4]. In marketing, political, and education studies, these advances have been rapidly leveraged to influence consumer and citizen behavior but have been rarely used in public health communication [5–9]. Our research focuses on leveraging recent advances in theory-grounded social network analytics that suggest unique ways to implement, monitor, and evaluate public health awareness campaigns on social media.

Over the last five years, several groups have implemented Twitter awareness campaigns focused on breast cancer [10], skin cancer [11], mental health [12], cardio-oncology [13], HPV vaccination [14], and breastfeeding [15, 16]. Independent of the varied content, audience, and duration across the studies, the methods used to assess campaigns were limited to one or several of the following approaches: content analysis of tweets; survey-based assessment of users’ knowledge; and/or quantitative analysis of Twitter Analytics metrics. These ubiquitous methods provide important information about individual users, such as their level of engagement with the campaign and their perspectives. However, these methods offer limited insight about the interactions among users and the resultant *social networks* (groups of people who interact together), which are known to influence knowledge diffusion, uptake, and decision making in public health [6, 17, 18].

Studies on social network theory (SNT) have shown how individuals are embedded in social relations and that their interactions may have consequences for individual and collective health behaviors [17, 18]. These interactions can be studied using SNT-based methods, which can be used for face-to-face relationships and for those happening online. We have recently applied these methods to understand exchanges in public health areas on Twitter using communications about breastfeeding as an exemplar case [19, 20]. We were able to identify unique communities (clusters of users) who discussed breastfeeding around the world and the specific influencers who dominated the conversations. These influencers were identified based on actual online interactions in real-time rather than solely on the number of followers, which is commonly reported. We also studied how "COVID and breastfeeding" World Health Organization guidelines were diffused on Twitter and characterized the extent of influence from the scientific community on messaging [21].

One of the remaining gaps in the literature and the next step in this work is establishing the potential of SNT-based metrics in better understanding public health campaigns. In this study, our goal is to showcase how a selection of SNT-based metrics, not fully established in public health communication research, can suggest unique approaches to implement, monitor, and evaluate awareness campaigns on Twitter. By using the "World Breastfeeding Week 2020 (WBW)" campaign as an example, we will review SNT-based metrics and approaches, apply them to the campaign’s Twitter data set, and report findings and implications to support future breastfeeding campaigns specifically and public health campaigns broadly. World Breastfeeding Week (WBW) is an annual and global awareness campaign (August 1–7) coordinated by the World Alliance for Breastfeeding Action (WABA) to inform, anchor, engage, and galvanize action on breastfeeding and related issues [22]. The 2020 campaign theme was "Support Breastfeeding for a Healthier Planet" to focus on the impact of infant feeding on the environment/climate change and the imperative to protect, promote and support breastfeeding for the health of the planet and its people [22].

## Methods

### a) Data collection

We accessed Twitter’s application programming interface using a dedicated server as previously described, complying with the terms and conditions for Twitter [19, 20]. We collected all tweets (content, sender, and date) that included at least one of the WBW-related hashtags (Table 1) starting July 30, 2020, and ending one month later based upon no more hashtag activity. We also collected profile information of all users associated with these tweets, such as their profile description, number of followers, and profile pictures. We used the same data collection approach during the six months prior to the campaign to capture all tweets and associated user data that included breastfeeding-related hashtags (Table 1). These specific hashtags were chosen based on our previous research that identified commonly-used hashtags in this space [19–21]. With no previous data available from last year’s campaign, we chose a six-month period as a conservative period to prevent covering tweets related to the previous year’s campaign. The six-month pre-campaign data reflects baseline activity related to breastfeeding discussions on Twitter and serves as the most up-to-date reference database to compare with campaign data. Because we collected all data in such a manner that the identity of subjects cannot readily be ascertained, directly or through identifiers linked to the subjects, the Institutional Review Board exempted this research from review.

**Table 1:** Hashtags used to capture tweets related to the campaign as well as breastfeeding-related conversations during the six months prior to the 2020 World Breastfeeding Week campaign.

Campaign hashtags	Breastfeeding-related hashtags
#WorldBreastfeedingWeek	#breastfeed
#WorldBreastfeedingWeek2020	#breastfeeding
#WBW	#normalizebreastfeeding
#WBW2020	#breastmilk
#BreastfeedingWeek	#breastfeedingsupport
#BreastfeedingWeek2020	#breastfeedingmoms

### b) Social Network Analysis (SNA)

We used Social Network Analysis (SNA) to visualize and describe user interactions at scale [23, 24]. SNA allows the description of patterns of social relationships that exist between people in a social network, such as identifying the role(s) of particular individuals within a network as well as the larger social infrastructure in which individuals interact and communicate [25, 26]. First, we constructed the social network maps for the WBW and pre-campaign databases using the open-source data visualization and exploration software GEPHI [27]. This software package can import network data and assist in exploring and mapping network structures for ease of visualization. In the case at hand, we considered as a connection, which has also been referred to as an edge or a link, every "retweet" or "mention" between two users [19, 20]. To identify whether the WBW data represent a single global conversation or many distinct smaller conversations, we analyzed groups of users using modularity clustering, which extracted the largest distinct communities [28]. Within one community, individuals communicate with each other more frequently than with individuals outside their community.

To identify influencers who dominated the WBW conversations disproportionally, we calculated outdegree (i.e., mentioning or retweeting someone), indegree (i.e., being mentioned or retweeted), overall degree (i.e., sum of in- and outdegree), and betweenness (i.e., how often someone is “in-between” two other users, for example, by retweeting someone and mentioning another person in the same tweet) for each user and selected the top 0.25% of users for each metric as we have done in previous work [29]. This work suggests a different form of "influence" than what is traditionally used in social media studies, which primarily focuses on the number of followers. From a social network perspective, we are interested in influence from a social structural sense through examining an individual’s "location" in the social network. This approach identifies four different types of influencers: transmitters, transceivers, transcenders, and traders (Table 2) [30–33]. Differences in influencer types between databases were determined by the Chi-Square test using SPSS (*P*<0.05 as statistical significance).

**Table 2:** Description of type of influencers, 2020 World Breastfeeding Week Campaign.

Type of influencers	Influencer description	Top 0.25% by metric	Metric description
Transmitters	Those who send the highest volume of tweets	Outdegree	number of out-going tweets, e.g., mentioning or retweeting others
Transceivers	The most mentioned users or those whose tweets are the most retweeted	Indegree	number of incoming tweets, e.g., being mentioned or retweeted
Transcenders	Those who send the highest volume of tweets and at the same time are the most mentioned and retweeted	Overall degree	Both in- and out-going activity
Traders	Those who bridge otherwise disconnected individuals	Betweenness	number of times a user sits between otherwise disconnected others

### c) Content analysis and topic modeling

To understand the background of the identified influencers, we used inductive qualitative coding [34] to categorize profiles into two categories: Scientific Community (*SC)* (academics, researchers, health care practitioners, and non-governmental agencies), or *members of the general public (GP)*. Coding was completed by two independent researchers after careful examination of influencers’ profiles and tweeting history, as well as verifying claimed credentials using websites such as those of academic institutions, governmental and non-governmental agencies, hospitals, and clinics as we have done in other published studies [19–21].

Finally, we conducted a bibliometric analysis of the tweets using topic modeling to determine what topics users discussed when using the campaign hashtags [35]. Bibliometric analysis is increasingly promoted as a valuable methodological tool to map what is being contributed and shared in large text corpora [36]. We employed latent dirichlet allocation using the Gibbs sampling algorithm to identify the underlying topical structure [37, 38]. By first analyzing the underlying topical structure of big textual datasets and then describing qualitatively the identified topics that best reflect the underlying communication flows, we were able to map the various topics being discussed using WBW hashtags. We assumed that topics that do not relate to the science of breastfeeding might be an indication of conversations that are less from the SC and more from the GP. Both social network analysis and bibliometric analysis were also conducted on the six-month breastfeeding database.

## Results

### 1. The WBW campaign attracted a high volume of inter-connected users

Within a period of three weeks only (July 30 to August 20, 2020), the campaign engaged almost three times the number of users (*n* = 29,958) who tweeted about breastfeeding in the last six months (*n* = 10,694). The number of tweets related to the campaign (*n* = 9,999) reached 70% of all breastfeeding-related tweets shared in the last six months (*n* = 14,213). This is equivalent to an average of 476 campaign-related tweets/day compared to a prior average of 79 tweets/day with general breastfeeding hashtags.

**Fig 1:**
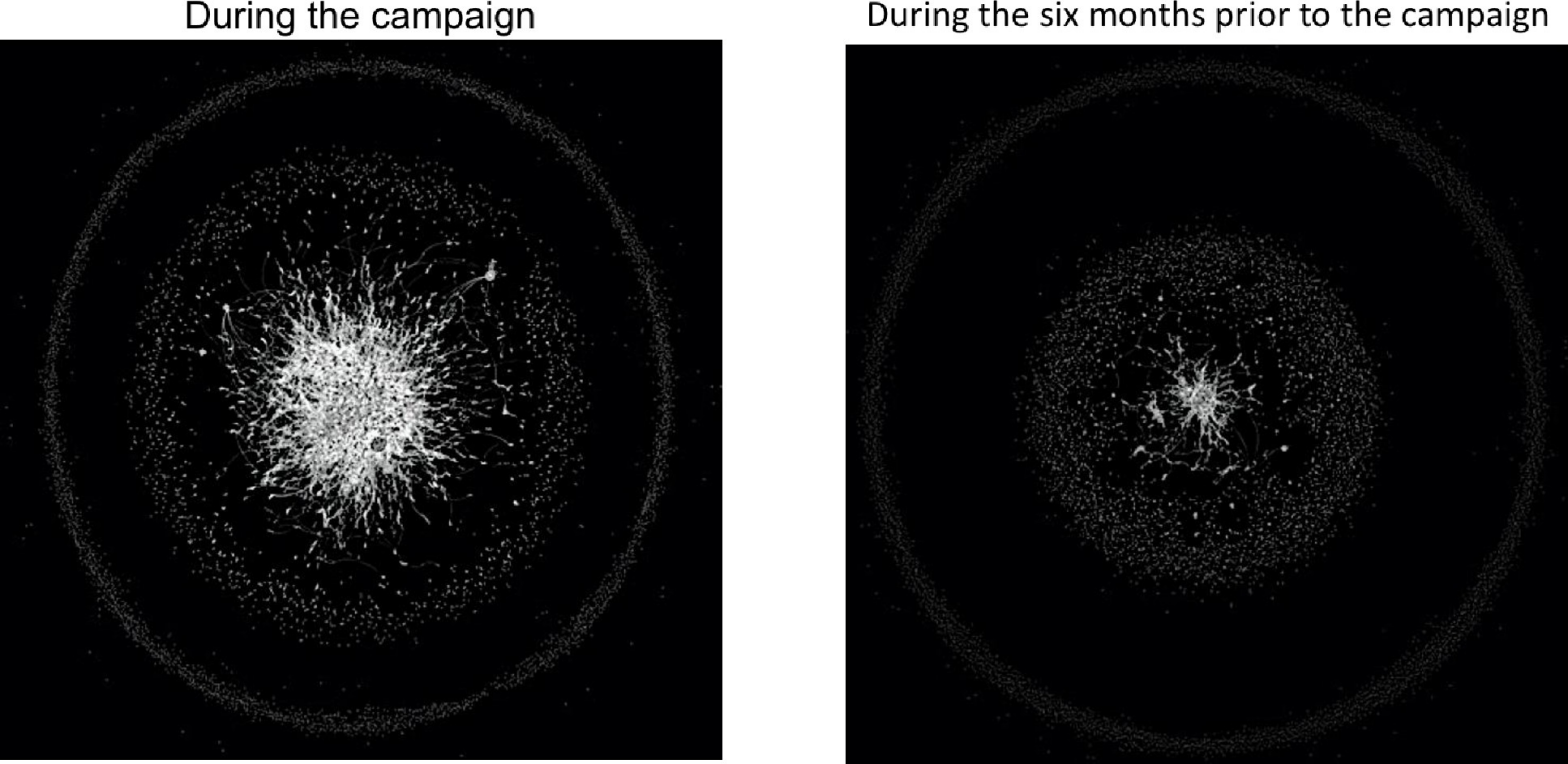
Social network maps. Each dot (node) represent a unique individual that tweeted to the network and the lines (edges) between the nodes reflect exchanged tweets (mentions and retweets). During the campaign, *n* = 29,958; Six-month data, *n* = 10,694.

To begin to understand the dynamic interactions between users during the campaign, we developed social network maps (Fig 1). The maps suggest a denser core of users with more inter-connected relationships during than before the campaign. Indeed, we found 46,161 edges (an edge or a link describes a connection between two users who retweet or mention each other) created during the campaign compared to only 10,662 edges before the campaign.

### 2. Users typically clustered in communities based on geographical location, professional background, and personal interests

**Fig 2:**
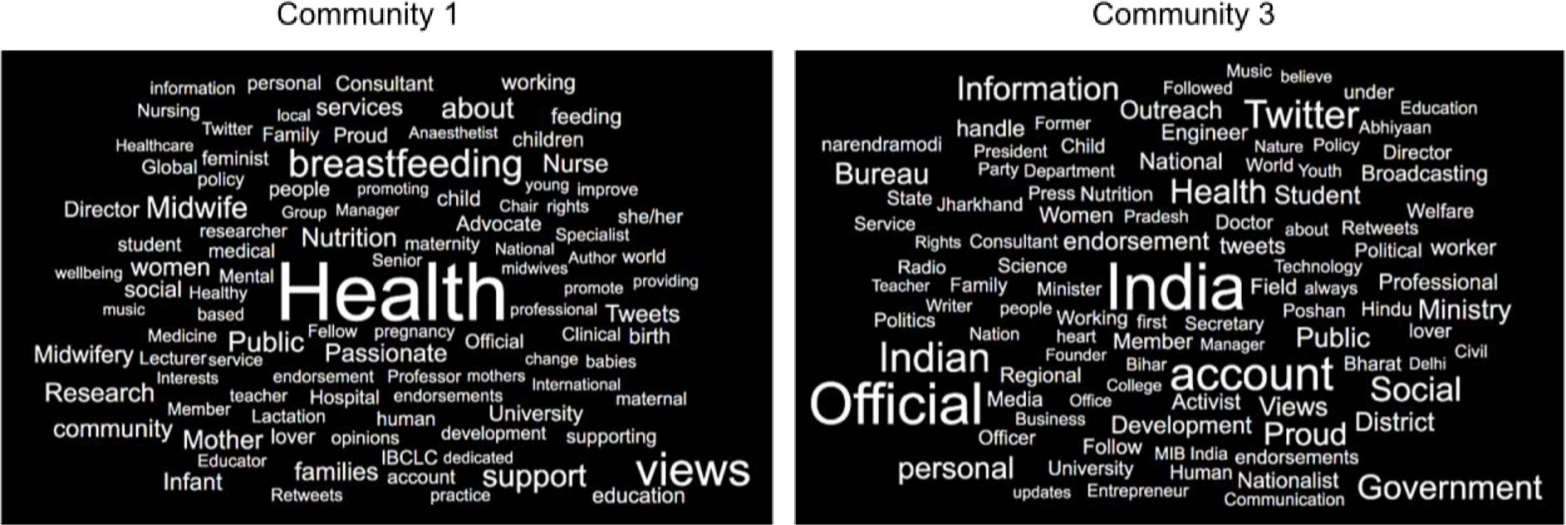
Profile description word clouds for users in Community 1 and 3 of the campaign network.

Next, we conducted modularity clustering that clustered users based on whom they interacted with more frequently than others in the network (S1 Fig). By segmenting the campaign network into communities based on real-time user behavior instead of our own a priori defined groupings, we identified the ten largest communities that accounted for almost 50% of the entire network (S2 Fig). To demonstrate the potential added value in identifying communities, we randomly selected Community 1 (12.7% of the total network) and Community 3 (6.7%) as examples and created word clouds based on the most frequently used texts in their Twitter profile descriptions (Fig 2) and locations (S3 Fig). Community 1 members appeared largely interested in and passionate about health as well as breastfeeding; They self-identified as having mostly scientific or clinical professions such as in medicine, midwifery, research, or lactation consultancy. While Community 1 members spanned many countries, including the United States and England, Community 3 members were predominately located in India, affiliated themselves with government-specific positions, and reported interests in country-wide politics. Therefore, by segmenting and analyzing social networks, unique insights into group formation/cohesion may open up opportunities for more targeted and informed campaign strategic decisions both cross-sectionally and over time.

### 3. Members of the general public reflected different profiles of influence from the scientific community

Another piece of information that may help inform campaign decisions is which influencers dominate the space of interest and what their characteristics are. We found the majority of *transcenders* (Those who send the highest volume of tweets and who are the most mentioned and retweeted; i.e., having high indegree and outdegree) during the campaign were from the SC, a finding largely driven by the significantly higher number of SC transceivers (indegree) during than before the campaign (*P*<0.001, Table 3). Put within the context of user behavior, the data suggests that influencers from the SC were not tweeting significantly more than they did before the campaign (*P* = 0.135). However, they were being mentioned or retweeted by others significantly more frequently during the campaign period. *Traders* (actors that connect otherwise disconnected others), on the other hand, tended to include a lower proportion of users from the SC during the campaign (*P* = 0.051), which suggests that the campaign may have mobilized the GP to use the campaign hashtags and then connect otherwise disconnected users on Twitter.

**Table 3:** Differences in the percentage of Twitter influencers from the scientific community between the campaign network and the six-months network; 2020 World Breastfeeding Week.

Influencer type	n (%)	P-value
**Transmitters**		0.135
Campaign	58 (77.3)	
Pre-campaign	47 (66.2)	
**Transceivers**		<0.001
Campaign	67 (88.2)	
Pre-campaign	37 (52.9)	
**Transcenders**		<0.001
Campaign	68 (90.7)	
Pre-campaign	41 (62.1)	
**Traders**		0.051
Campaign	61 (81.3)	
Pre-campaign	70 (92.1)	

%, percentage of members from the scientific community out of the top 75 users (top 0.25% by influence metric in campaign database); Pre-campaign: during the last six months prior to the campaign; P-value determined by Chi-Square test for differences between campaign and pre-campaign % SC.

### 4. Topics were connected to the main theme of the campaign but also reflected larger health and societal issues

To identify the commonly discussed themes when users tweeted the campaign hashtags, we employed topic modeling and identified seven topics describing Twitter discussions. While Topics 1 and 2 focused on raising awareness about the 2020 campaign theme (i.e., the role of breastfeeding in sustaining the planet), Topic 3 centered around the broader health benefits of breastfeeding (S1 Table). Topics 4 and 5 were tangentially related to the main campaign theme as they focused on breastfeeding clinical guidelines, both about and beyond COVID, and support systems available for mothers to continue breastfeeding. Finally, Topic 6 highlighted the expansion of tweets beyond the English language, and Topic 7 tied the campaign to topics of social and racial justice in the United States, such as the Black Lives Matter movement. Combined, these results highlight that the WBW campaign attracted a dynamic and identifiable network of users and influencers from both the SC and the GP from around the world who engaged in discussions–not only about the campaign’s 2020 theme but also about physical and societal health more broadly.

## Discussion

In this study, we reported that the 2020 WBW campaign engaged a global network of approximately 30,000 users who formed unique geographically-diverse and interest-driven communities and who discussed the campaign within and beyond the scope of its original theme. Additionally, we found that influencers from the SC and the GP served different roles: the role of "authority" or "prestige" for the SC by being so frequently mentioned and the role of "weaving" or connecting users together by the GP. Collectively, this information can help inform strategic decisions, both cross-sectionally and prospectively, related to the campaign’s various phases from planning to evaluation. More broadly, by using WBW as an exemplar case, we demonstrate how applying a selection of SNT-based metrics can generate novel insights into a social media-based public health campaign.

The proposition that the virtual world can be socially mapped and measured to provide insights and action is a quickly evolving space [39, 40]. Precedents of effective SNT-based applications for online data have been set, predominately in the field of education and policy and, more recently, in public health [19–21, 39]. An ample amount of data from these studies suggests social media is the "backbone" of the Internet, where all types of media, links, ideas, opinions, and tools are shared among social networks. Particularly on Twitter, communities within networks arise organically rather than as organized by external sources such as on-ground organizations. Taken together, key findings from research in other disciplines seem to be consistent with our findings, suggesting public health campaigns can equally benefit from SNT-based metrics and approaches grounded in the growing field of Network Science.

In conducting this research, we did not intervene (e.g., tweet using the campaign hashtags or reach out to influencers to modify behavior), nor did we set a list of metric benchmarks to evaluate the campaign. Our role as researchers was to explore and describe the interactions taking place during the campaign under ’real world’ conditions as a way to establish a baseline and test out our ideas around the use of SNT in public health campaigns. Making conclusions about the campaign’s effectiveness is beyond the scope of our work and is left to the campaign organizers. However, our findings highlight several questions that may guide future campaign decisions, such as: 1) If the campaign goal is to raise awareness, to what degree are target audiences being reached; 2) Which topics are actually flowing through the network and were those intended; 3) To what extent are influencers key to moving messages and sharing perspectives; 4) Are campaign goals more likely to be achieved if users engage in one global conversation focused only on the campaign’s main themes or a variety of topics; 5) Are messages being tailored to different sub-groups of users based on defined individual characteristics (e.g., demographics, professional background, location) and what is the uptake of those messages; 6) To what degree is a campaign more likely to be successful if its duration is expanded beyond a one-week period, and would it be of value to monitor the evolution of communities in real-time and the topics discussed; as well as 7) With real-time SNT analytics available, how might a campaign conduct short-cycle testing to evaluate the effects of refocusing tweets or seeding key influencers with messages related to the campaign? There is a multitude of key questions and strategic decisions that might be made with access to SNT-grounded data along the trajectory of a campaign.

While proof-of-concept for using SNT-based analytics in public health research now enjoys a growing body of work by our group and others [17–19], it is important to address a range of limitations in future studies. First and as previously suggested [41, 42], coupling the SNT approach with methods that assess behavior change or public health outcomes over time might provide a more robust understanding of social networks’ role in a campaign’s success. One example is to conduct a network-based intervention in which researchers identify and seed influencers with campaign messages and then measure whether this approach results in higher diffusion of information and improved breastfeeding practices among an experimental group of parents compared to a control group with typical Twitter activity. Second, most user profile information collected from social media platforms are self-reported, and, thus, strategic decisions should be based on general trends in big data in addition to individual user data, which alone could be misleading. Third, by analyzing the datasets cross-sectionally, we might have overlooked important nuances in the composition of communities and influencers over time, which would be best determined using a prospective study design. Particularly in the reference six-months pre-campaign database, it would be interesting to explore how the evolution of events in 2020, such as the declaration of the COVID pandemic and the release of various health and safety guidelines, have impacted the "typical" communication around breastfeeding on Twitter. For example, by sampling Twitter data by time points and identifying influencers when COVID hashtags were or were not used, it is possible to determine whether new influencers emerged during the pandemic and whether their contribution to the WBW campaign discussion was constructive. Additionally, it would be important to expand the analysis to Facebook, Instagram, and other social media platforms, as well as to different modes of communication, including WhatsApp, to ensure a more comprehensive assessment of reach as users may share links and, therefore, knowledge across platforms (e.g., from Twitter to Facebook). Finally, it would be interesting to determine whether the campaign attracted a higher % of GP compared to the prior six-month period. In the future, this can be addressed by extending the content analysis of users’ profile description to all users in both datasets, not only for influencers in the campaign (*n* = 75).

Relying only on static metrics of user engagement, such as the number of likes and followers, might not be sufficient to comprehensively inform public health stakeholders about campaign reach and effectiveness. The analytical approach and findings from the WBW case study have public health implications: Analyzing data with a social network lens provides the opportunity for a deeper understanding of real-time interactions and the relationships that are being formed during online public health campaign activities, which are known to impact knowledge dissemination, uptake and potentially health behavior. In the case of WBW, moving forward, our analysis suggests that improve targeting could enhance geographic coverage and audience diversity and interactions among them.

## Supporting information

S1 Fig Social Network Maps by communities. Each dot (node) represents a unique user that tweeted to the network, and the lines (edges) between the nodes reflect exchanged tweets (mentions and retweets). The size of the nodes is based on their overall degree centrality. The color of the nodes represents the community to which they have been assigned by the Louvain community algorithm. During the campaign, *n* = 29,958; six-months data, *n* = 10,694. 2020 World Breastfeeding Week Campaign.
(DOCX)

S2 Fig The top ten communities ranked by size as a percentage of the entire campaign network. Communities were identified using modularity clustering (20). On the x-axis, the number denotes rank of community; for example, 2 = community with the second largest number of users. 2020 World Breastfeeding Week Campaign.
(DOCX)

S3 Fig Geographical location word clouds for users in Community 1 and 3 of the campaign network. 2020 World Breastfeeding Week Campaign.
(DOCX)

S1 Table Common topics discussed on Twitter during the 2020 World Breastfeeding Week campaign. 2020 World Breastfeeding Week Campaign.
(DOCX)
