# Supplementary figures and images for: Real-time Twitter interactions during World Breastfeeding Week: A case study and social network analysis
Source: PLoS One. 2021 Mar 29;16(3):e0249302. doi: 10.1371/journal.pone.0249302 (PMC8007060; doi:10.1371/journal.pone.0249302)

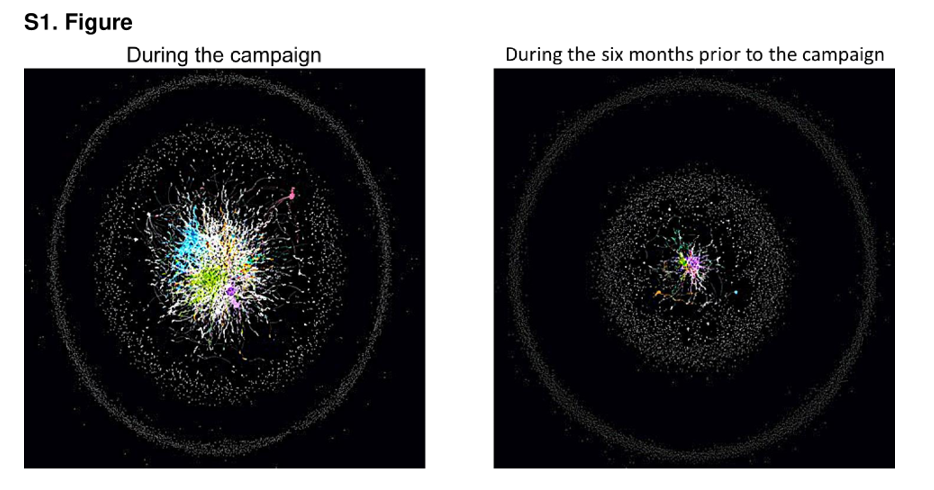

Supplement: S1 Fig — Each dot (node) represents a unique user that tweeted to the network, and the lines (edges) between the nodes reflect exchanged tweets (mentions and retweets). The size of the nodes is based on their overall degree centrality. The color of the nodes represents the community to which they have been assigned by the Louvain community algorithm. During the campaign, n = 29,958; six-months data, n = 10,694. 2020 World Breastfeeding Week Campaign. (DOCX) [file pone.0249302.s001.docx]

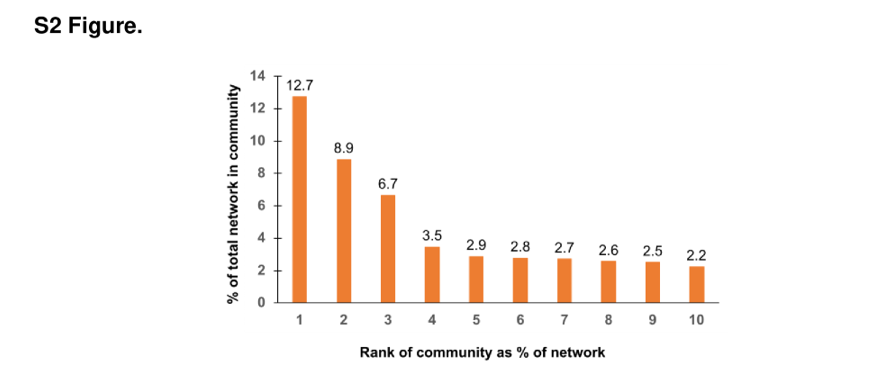

Supplement: S2 Fig — Communities were identified using modularity clustering (20). On the x-axis, the number denotes rank of community; for example, 2 = community with the second largest number of users. 2020 World Breastfeeding Week Campaign. (DOCX) [file pone.0249302.s002.docx]

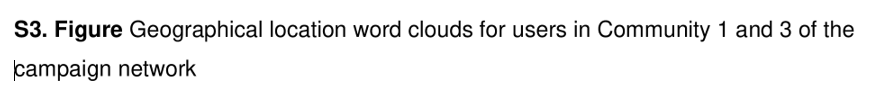

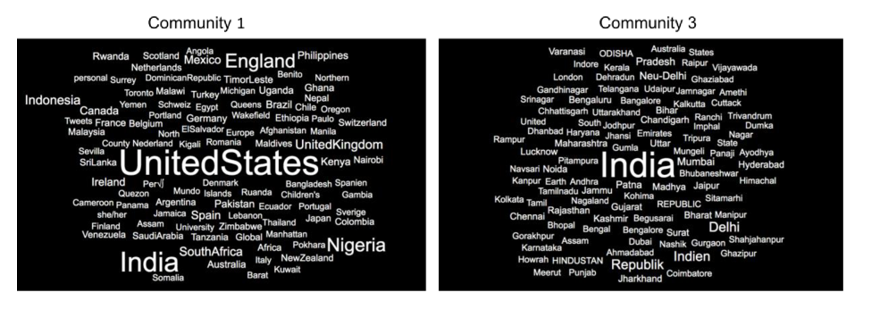

Supplement: S3 Fig — 2020 World Breastfeeding Week Campaign. (DOCX) [file pone.0249302.s003.docx]

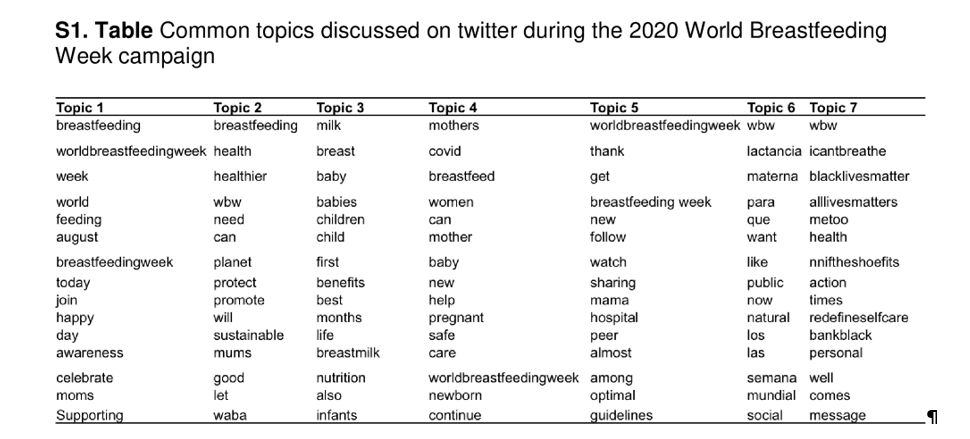

Supplement: S1 Table — 2020 World Breastfeeding Week Campaign. (DOCX) [file pone.0249302.s004.docx]
